# β-Cell-Specific *Mafk* Overexpression Impairs Pancreatic Endocrine Cell Development

**DOI:** 10.1371/journal.pone.0150010

**Published:** 2016-02-22

**Authors:** Ahmed M. Abdellatif, Hisashi Oishi, Takahiro Itagaki, Yunshin Jung, Hossam H. Shawki, Yukari Okita, Yoshikazu Hasegawa, Hiroyuki Suzuki, Salah E. El-Morsy, Mesbah A. El-Sayed, Mahmoud B. Shoaib, Fumihiro Sugiyama, Satoru Takahashi

**Affiliations:** 1 Department of Anatomy and Embryology, Faculty of Medicine, University of Tsukuba, Tsukuba, Ibaraki, Japan; 2 Department of Anatomy and Embryology, Faculty of Veterinary Medicine, Mansoura University, Mansoura, Egypt; 3 Laboratory of Animal Resource Center, Faculty of Medicine, University of Tsukuba, Tsukuba, Ibaraki, Japan; 4 Life Science Center, Tsukuba Advanced Research Alliance (TARA), University of Tsukuba, Tsukuba, Ibaraki, Japan; 5 Department of Experimental Pathology, Faculty of Medicine, University of Tsukuba, Tsukuba, Ibaraki, Japan; 6 International Institute for Integrative Sleep Medicine (WPI-IIIS), University of Tsukuba, Tsukuba, Ibaraki, Japan; Broad Institute of Harvard and MIT, UNITED STATES

## Abstract

The MAF family transcription factors are homologs of v-Maf, the oncogenic component of the avian retrovirus AS42. They are subdivided into 2 groups, small and large MAF proteins, according to their structure, function, and molecular size. MAFK is a member of the small MAF family and acts as a dominant negative form of large MAFs. In previous research we generated transgenic mice that overexpress MAFK in order to suppress the function of large MAF proteins in pancreatic β-cells. These mice developed hyperglycemia in adulthood due to impairment of glucose-stimulated insulin secretion. The aim of the current study is to examine the effects of β-cell-specific *Mafk* overexpression in endocrine cell development. The developing islets of *Mafk*-transgenic embryos appeared to be disorganized with an inversion of total numbers of insulin+ and glucagon+ cells due to reduced β-cell proliferation. Gene expression analysis by quantitative RT-PCR revealed decreased levels of β-cell-related genes whose expressions are known to be controlled by large MAF proteins. Additionally, these changes were accompanied with a significant increase in key β-cell transcription factors likely due to compensatory mechanisms that might have been activated in response to the β-cell loss. Finally, microarray comparison of gene expression profiles between wild-type and transgenic pancreata revealed alteration of some uncharacterized genes including *Pcbd1*, *Fam132a*, *Cryba2*, and *Npy*, which might play important roles during pancreatic endocrine development. Taken together, these results suggest that *Mafk* overexpression impairs endocrine development through a regulation of numerous β-cell-related genes. The microarray analysis provided a unique data set of differentially expressed genes that might contribute to a better understanding of the molecular basis that governs the development and function of endocrine pancreas.

## Introduction

Diabetes mellitus is a group of metabolic diseases characterized by hyperglycemia resulting from defects in insulin secretion, insulin action, or both, which eventually leads to a series of complications in various organs [1]. Type 1 diabetes (T1D) results from the destruction of β-cells by a β-cell-specific autoimmune reaction. In type 2 diabetes (T2D), the peripheral tissues are resistant to insulin action and the disease is often accompanied by obesity and hyperlipidemia. For decades, several approaches have been developed for the treatment of diabetes including insulin-secretion stimulants, improving insulin preparations, and islet transplantation, yet many unexamined avenues of research remain [2].

During pancreatic development, a subset of the pancreatic epithelial cells starts to express the proendocrine factor *Ngn3* and gives rise to all types of endocrine cells [3–6]. The hormone-expressing cells are produced during 2 sequential stages, the primary and secondary transitions. The primary transition begins before E13.5, and is characterized by an appearance of hormone+ cells that are not fully functional. During the secondary transition starting from around E13.5, the differentiating endocrine cells expand markedly, then migrate into mesenchyme, and eventually aggregate to form Langerhans islets [7]. The expression of a cascade of different transcription factors stimulates the differentiation into distinct endocrine lineages. α-cell-related transcription factors include *Mafb*, *Nkx2*.*2*, *Pax6*, *Foxa2*, *Pou3f4*, and *Arx*, whereas β-cell differentiation is controlled by *Pdx1*, *Mafb*, *Pax4*, *Pax6*, *Nkx2*.*2*, and *Nkx6*.*1* [8].

The MAF family transcription factors belong to the activator protein 1 (AP1) superfamily of basic leucine zipper (bZIP) proteins. It derives its name from v-Maf—the oncogenic component of the avian retrovirus AS42 that was originally isolated from chicken musculoaponeurotic fibrosarcoma [9]. The MAF family is subdivided into 2 groups according to their molecular size: the small MAF proteins comprising MAFG, MAFF, and MAFK; and large MAF proteins, including MAFA, MAFB, c-MAF, and NRL. All MAF proteins contain basic leucine zipper domain that allows DNA binding. Compared to the other bZIP proteins, MAF proteins can recognize a longer palindromic sequence of DNA (Maf-recognition element, MARE) [10–12]. Increasing numbers of studies on endocrine development reveal that the expression of large MAF proteins is tightly regulated in a spatiotemporal manner [13–15]. *Mafb* gene knockout (*Mafb*^−/−^) mice show around a 50% reduction of α- and β-cell numbers at E18.5. In contrast, no developmental defects were observed in *Mafa* gene knockout (*Mafa*^−/−^) mice [16, 17]. Small MAF proteins are also found to display a complex expression pattern during embryogenesis [18]. They are able to form a homodimer or a heterodimer with other bZIP factors such as the cap’n’collar (CNC) family and play a role in many biological processes like hematopoiesis, neuronal function, and oxidative stress response [19–22]. Unlike the large MAF proteins, the small MAF proteins lack a transactivation domain and when they are expressed in large amounts, the homodimeric proteins compete with the binding of large MAF proteins to the cis-element of target genes at MARE sites, resulting in a dominant-negative effect [23].

Our previous studies demonstrated that β-cell-specific *Mafk* transgenic (*Mafk*-Tg) mice exhibited hyperglycemia due to an impaired insulin secretion during early postnatal life [24]. When these mice are crossed with *Mafa*^−/−^ mice, the double mutants display destructive β-cell development and an overt diabetic phenotype with typical characteristics of human diabetic nephropathy [25]. The aim of the current study was to characterize and evaluate the impact of *Mafk* overexpression on the genetic pathways governing β-cell development using *Mafk*-Tg and *Mafa*^−/−^;*Mafk*-Tg mice embryos. In the *Mafk*-Tg mutants we observed abnormalities in β-cell development and islet morphogenesis along with a reduction of β-cell proliferation. In addition, we also performed a microarray analysis in order to investigate the factors that contribute to the phenotypic alteration in the *Mafk*-Tg mice at E15.5, to gain insights into the mechanisms controlling endocrine cell development and function.

## Materials and Methods

## Mice

The mice were maintained in specific pathogen-free conditions, in the Laboratory Animal Resource Center at the University of Tsukuba. This study was carried out in strict accordance with the recommendations in the Guide for the Care and Use of Laboratory Animals of the National Institutes of Health. The protocol was approved by the Committee on the Ethics of Animal Experiments of the University of Tsukuba (Permit Number: 14–049). All mice were euthanized with carbon dioxide gas, and all efforts were made to minimize suffering. The generations of *Mafa* knockout (*Mafa*^−/−^) mice, transgenic mice expressing *Mafk* under the control of rat *Insulin 1* promoter (*Mafk*-Tg), R26GRR mice, and *Ins1*-Cre25 mice have previously been described [16, 24, 26, 27]. Double mutant *Mafa*^−/−^;*Mafk*-Tg mice were generated by mating *Mafa*^+/−^ females with *Mafa*^+/−^;*Mafk*-Tg males. DNA extraction and embryo genotyping were performed by NaOH extraction methods from tails as previously described [16, 24].

### Immunohistochemistry

The embryos were collected, washed in cold PBS, fixed in 4% PFA, and then embedded in paraffin. Immunohistochemistry was performed on 5-μm paraffin sections according to the standard histological methods. The sections were blocked in appropriate serum for 1 hour and incubated overnight at 4°C with the following primary antibodies: guinea pig anti-insulin (ab7842, 1:1000, Abcam, Cambridge, UK), rabbit anti-glucagon (RAG-06P, 1:2000, Linco Research, St. Charles, MO, USA), guinea pig anti-glucagon (M182, 1:4000, Takara, Kyoto, Japan), rabbit anti-aristaless-related homeobox (gift from Drs. Kitamura and Morohashi; 1: 250) [28], rabbit anti-Ki67 (NCL-Ki67p, 1:500, Novocastra, Newcastle, UK) and rabbit anti-PHH3 (ab5176, 1:500, Abcam). The antigens were visualized using the appropriate secondary antibodies conjugated with Alexa Fluor 350, 488, or 594 (1:1000, Life Technologies, Gaithersburg, MD, USA). All sections were examined using a fluorescence microscope (BZ-9000, Keyence, Tokyo, Japan). For cell counting experiments, serial sections spanning the entire pancreas were collected at a 100-μm intervals and immunostained. A total number of 25 sections were used per pancreas. The total numbers of immunoreactive cells were quantified using ImageJ 1.48 software (NIH, Bethesda, Maryland, USA).

### Measurement of total insulin contents

The whole pancreas was collected from embryos at both E15.5 and E18.5. The total insulin content was determined after extraction with acid-ethanol. Insulin levels were detected using a mouse insulin ELISA kit (Morinaga, Yokohama, Japan).

### Luciferase assay

The *Mafk*-expression plasmid and RIPII-251 reporter plasmid have been previously described [24, 29]. The *Mafb* cDNA was subcloned into the pcDNA3.1-FLAG expression vector, and these plasmids were transfected into NIH3T3 cells using FuGENE 6 transfection reagent (Roche, Indianapolis, IN, USA). The total amount of DNA was adjusted by cotransfection of pcDNA3.1-FLAG plasmid with *Mafk*-expression plasmids. Luciferase activities were determined by the Dual Luciferase Reporter Assay System (Promega) 48 hours after transfection.

### Chromatin Immunoprecipitation (ChIP) assay

NMuMG cells expressing FLAG-MAFK were prepared as previously described [30]. In short, the NMuMG cells were transfected with pCAGIP-FLAG-MAFK or mock plasmids. The cells were cross-linked with 1% formaldehyde at 37°C for 15 minutes, suspended in 500 μl of nuclear lysis buffer (1% SDS, 50 mM Tris-HCl, pH 8.1, 10 mM EDTA, 20,000 KIU/ml aprotinin, 1 μg/ml leupeptin), and sonicated. Soluble chromatin was diluted with 9 volumes of dilution buffer for immunoprecipitation (16.7 mM Tris-HCl, pH 8.1, 1.2 mM EDTA, 167 mM NaCl, 0.01% SDS, 1.1% Triton X-100, 20,000 KIU/ml aprotinin, 1 μg/ml leupeptin) and incubated with anti-FLAG antibody (M2, Sigma, St Louis, MO, USA) with end-over-end rotation at 4°C overnight followed by incubation with 25 μl of Dynabeads Protein A (Life Technologies) at 4°C for 1 hour. DNA was extracted from the Dynabeads by means of phenol-chloroform extraction. The PCR primers are described in Table 1.

**Table 1:** The primers used in this study.

Gene Name	Foreward (5`- 3`)	Reverse (5`- 3`)
*Arx*	TCCGGATACCCCACTTAGCTT	GACGCCCCTTTCCTTTAAGTG
*Chgb*	CCCTCAGCTCGACTTGAAAC	GCCGTCAGAACTCTCTGGTC
*Cryba2*	ACCAGCAAAGATGTGGGTTC	GGACTCTTCGAATGGACTGC
*Fam132a*	GCCAGATGATGGGTCCTCTA	TCGAAGTTCTGTGGCCTCTT
*Foxa2*	GAGCACCATTACGCCTTCAAC	AGGCCTTGAGGTCCATTTTGT
*G6pc2*	TGCGTCTGGTATGTCATGGT	TTCAAAGGCCTCGGCTACTA
*Gcg*	AGGGACCTTTACCAGTGATGT	AATGGCGACTTCTTCTGGGAA
*Gck*	TGGATGACAGAGCCAGGATGG	ACTTCTGAGCCTTCTGGGGTG
*Glut2*	AAGGATCTGCTCACATAGTCACT	TTGCAGCCAACATTGCTTTGA
*Hnf1a*	TATCATGGCCTCGCTACCTG	ACTCCCCATGCTGTTGATGA
*Hprt*	TTGTTGTTGGATATGCCCTTGACTA	AGGCAGATGGCCACAGGACTA
*Ins1*	GCCCTCTGGGAGCCCAAA	AGAGAGCCTCTACCAGG
*Ins2*	GCTTCTTCTACACACCCATGTC	AGCACTGATCTACAATGCCAC
*Ins promoter*	TGAAACAGTCCAAGG	ACTTTGCTGTTTG
*Kir6*.*2*	GTAGGGGACCTCCGAAAGAG	TGGAGTCGATGACGTGGTAG
*Mafa*	CACTGGCCATCGAGTACGTCA	CTTCACCTCGAACTTCATCAGGTC
*Mafb*	TGAATTTGCTGGCACTGCTG	AAGCACCATGCGGTTCATACA
*Mafk*	GAGAAGCTGGCTCGAGAGAA	CGGCTGAGAAGGGTACAGAG
*Neurod1*	ACAGACGCTCTGCAAAGGTTT	GGACTGGTAGGAGTAGGGATG
*Ngn3*	TCTCAAGCATCTCGCCTCTTC	ACAGCAAGGGTACCGATGAGA
*Nkx2*.*2*	CCGGGCGGAGAAAGGTATG	CTGTAGGCGGAAAAGGGGA
*Nkx6*.*1*	CAGACCCACGTTCTCTGGAC	TGACCTGACTCTCCGTCATCC
*Pax4*	TGGCTACACAGACAGCATTTAC	GCGCTTGTTATTCGCTGGTC
*Pax6*	AACAACCTGCCTATGCAACC	ACTTGGACGGGAACTGACAC
*PC1/3*	ATGGGCGGCGGAGATC	CCAATCTGACCCAAAAGGTCATAC
*PC2*	AATGACCCCTACCCATACCC	GAGGAGGCTTCGATGATGTC
*Pcbd1*	AGGCCGAGATGCTATCTTCA	ATATCCCGTTCCGAAAGACC
*Pdx1*	TTCCCGAATGGAACCGAGC	GTAGGCAGTACGGGTCCTCT
*Pou3f4*	CTCGCCGCACACTAACCAT	GCTCCAGCATACCGCTCAC
*Rbp4*	TTCTGTGGACGAGAAGGGTC	GTGCCATCCAGATTCTGCAG
*Rfx6*	TGTGAAGAACGAAAGCCACG	TGGAGAAATCGGTGGTGTCA
*Slc30a8*	ACTGATGCGGCTCATCTCTT	GATGCAAAGGACAGACAGCA
*Sur1*	CTGGTCCTCAGCAGCACAT	GGAACTCTTGGGACGAGACA
*Sytl4*	AGTCTGTGGTGATGAGGGTG	CCAGGTGGTCAATGTCCTCT
*Tmem27*	GAGCAATGGTGGCATTCTCC	ACTTCAGCTGCAGGAAGAGT

### Quantitative RT-PCR

The whole pancreas of E15.5 embryos were homogenized and total RNA isolated using the NucleoSpin RNA kit (Macherey-Nagel, Düeren, Germany). The cDNA was synthesized using a QuantiTect Reverse Transcription Kit (Qiagen, Hilden, Germany). Quantitative PCR reactions were carried out using a Thermal Cycler Dice Real Time System (Takara) with a SYBR Green PCR Master Mix (Takara). Expression of *Hprt* was utilized to analyze the relative gene expression of other genes. All primer sequences are listed in Table 1.

### Lineage tracing experiments

To test the possibility that β-cell specific *Mafk* overexpression induced β-cell transdifferentiation into α-cells during early stages of development, we crossed *Mafk*-Tg females with *Ins1*-Cre;R26GRR males, which express GFP ubiquitously before and tdsRed after Cre excision; thus we could label around 90% of the β-cell lineage [26]. The pancreata of *Mafk*-Tg;*Ins1*-Cre;R26GRR and control WT;*Ins1*-Cre;R26GRR mice were collected at both P0 and 4 weeks of age, fixed overnight in 4% PFA, dehydrated in sucrose and embedded in OCT compound (Sakura, Tokyo, Japan). Frozen sections were stained using anti-glucagon antibody in order to determine the cell fraction coexpressing both tdsRed and glucagon.

### Microarray experiment

Total RNA was isolated from 3 pairs of WT and *Mafk*-Tg pancreata at E15.5 using the NucleoSpin RNA kit (Macherey-Nagel). The total RNA was used to synthesize cRNA using the Ambion WT Expression Kit (Life Technologies). Fragmentation and labeling of cDNA were performed using the GeneChip WT Terminal Labeling and Control Kit (Affymetrix, Santa Clara, CA, USA). The hybridization cocktail containing fragmented and biotin-labeled cDNAs was transferred into GeneChip MoGene-1_0-st-v1 cassettes (Affymetrix), which were incubated at 45°C inside a hybridization oven by rotating them at 60 rpm for 17 hours. The GeneChip arrays were then washed and developed using the Hybridization Wash and Stain Kit (Affymetrix) in a Fluidics Station 450 (Affymetrix). The GeneChip arrays were read using the GeneChip Scanner 3000 (Affymetrix) and the image files were generated using the GeneChip Command Console (Affymetrix). Normalization and probe set summarization were performed using the Affymetrix Expression Console software. CEL files and normalized data were deposited into the NCBI GEO repository under the accession number GSE62834.

### Statistical analysis

Data were expressed as the means ± standard errors of the means and compared using an unpaired *t* test. Probability values of less than 0.05 were considered significant.

## Results

### β-cell-specific *Mafk* overexpression resulted in impaired endocrine cell development and an abnormal islet structure

**Fig 1:**
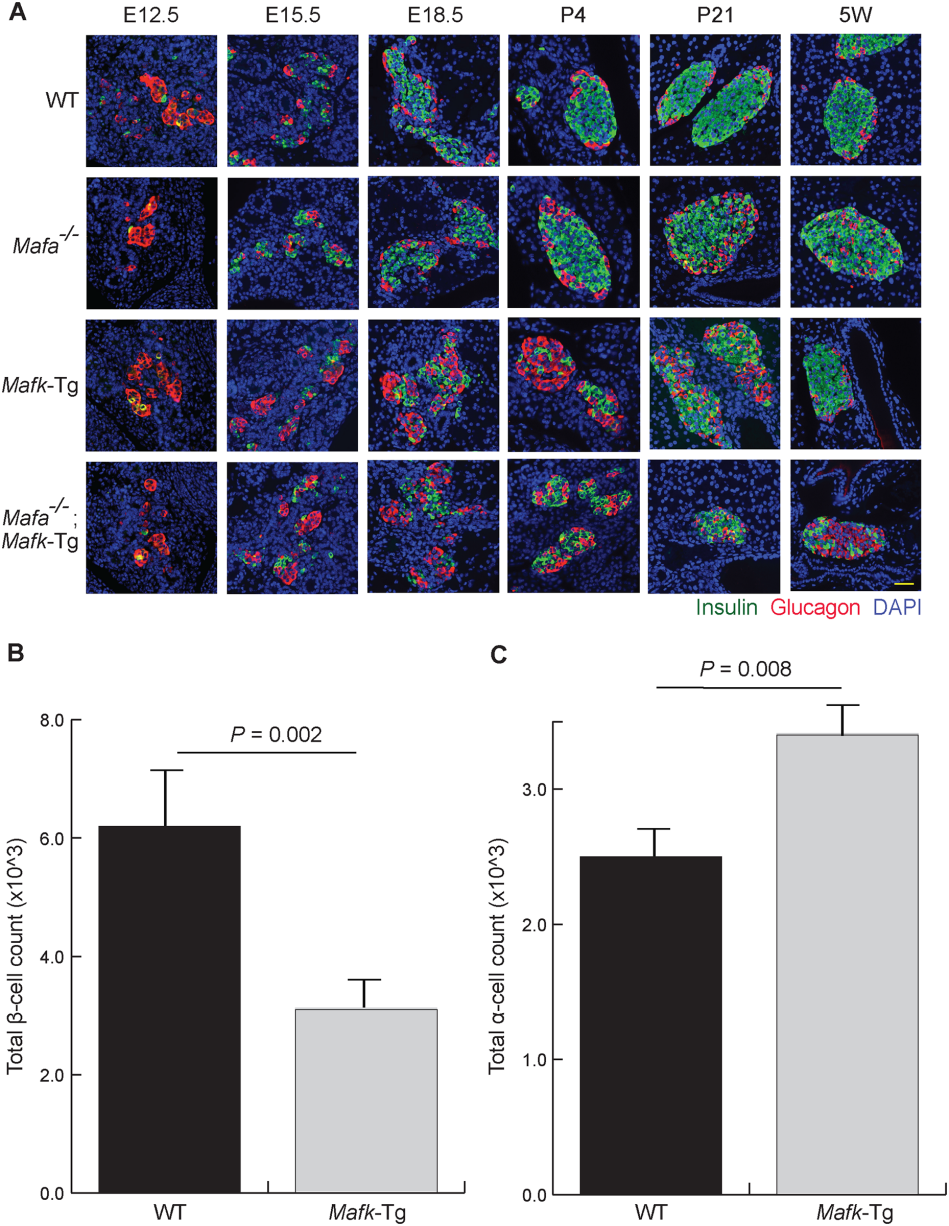
Transgenic *Mafk* overexpression altered the normal islet structure. (A) Immunohistochemical analysis of insulin and glucagon in wild-type (WT), *Mafa* knockout (*Mafa*^−/−^), *Mafk* transgenic (*Mafk*-Tg) and *Mafa*^−/−^;*Mafk*-Tg pancreata at embryonic day (E) 12.5, E15.5, E18.5, postnatal day (P)4, P21 and 5 weeks of age (5W). Scale bar = 40 μm. (B) The total β-cell count of WT (n = 3) and *Mafk*-Tg (n = 5) embryos at E18.5. (C) The α-cell count of WT (n = 3) and *Mafk*-Tg (n = 5) embryos at E18.5. The error bars represent the standard errors of the means.

In order to study the impact of β-cell-specific *Mafk* overexpression on endocrine development, especially during the primary and secondary transitions, we performed immunohistochemical staining of pancreas sections from WT, *Mafa*^−/−^, *Mafk*-Tg, and *Mafa*^−/−^;*Mafk*-Tg embryos (Fig 1A). At E12.5, the point at which the primary cells emerge (the first wave of hormone-expressing cells), all groups of mice showed comparable manifestations. They contained a few insulin positive cells and a few insulin/glucagon double positive cells (which are considered progenitor cells), while the majority of cells were glucagon-positive [31, 32]. At E15.5 and E18.5, the islets of WT and *Mafa*^−/−^ embryos displayed normal and comparable phenotypes, whereas *Mafk*-Tg and *Mafa*^−/−^;*Mafk*-Tg embryos exhibited similar structural patterns with reversed number of insulin+ and glucagon+ cells. These results indicate that *Mafa* is not involved in β-cell development either in the *Mafk*-Tg background or in the WT background. We also stained pancreatic sections at postnatal day (P) 4, P21, and 5 weeks of age (5W). The islets from WT mice at all periods appeared with a clear central core of insulin+ cells surrounded by a peripheral layer of glucagon+ cells, whereas *Mafa*^−/−^ mice developed an abnormal structure with many glucagon+ cells in the center of the islets in the early neonatal period [16]. The islets of *Mafk*-Tg mice were still displaying changes similar to their embryonic abnormal phenotype at P4 and P21, however at 5W the islet structure was apparently reverted to normal. As shown previously, in the islet of *Mafa*^−/−^;*Mafk*-Tg mice, the destructive changes were more severe compared to *Mafa*^−/−^ mice [25]. These results suggest that the expression of *Mafa* during the neonatal period compensates for the effect of *Mafk* overexpression in embryos, which is consistent with a previous report showing the functional significances of *Mafa* after birth [33]. Hereafter, our experiment was focused on the comparison of *Mafk*-Tg and control WT mice in each experiment.

**Fig 2:**
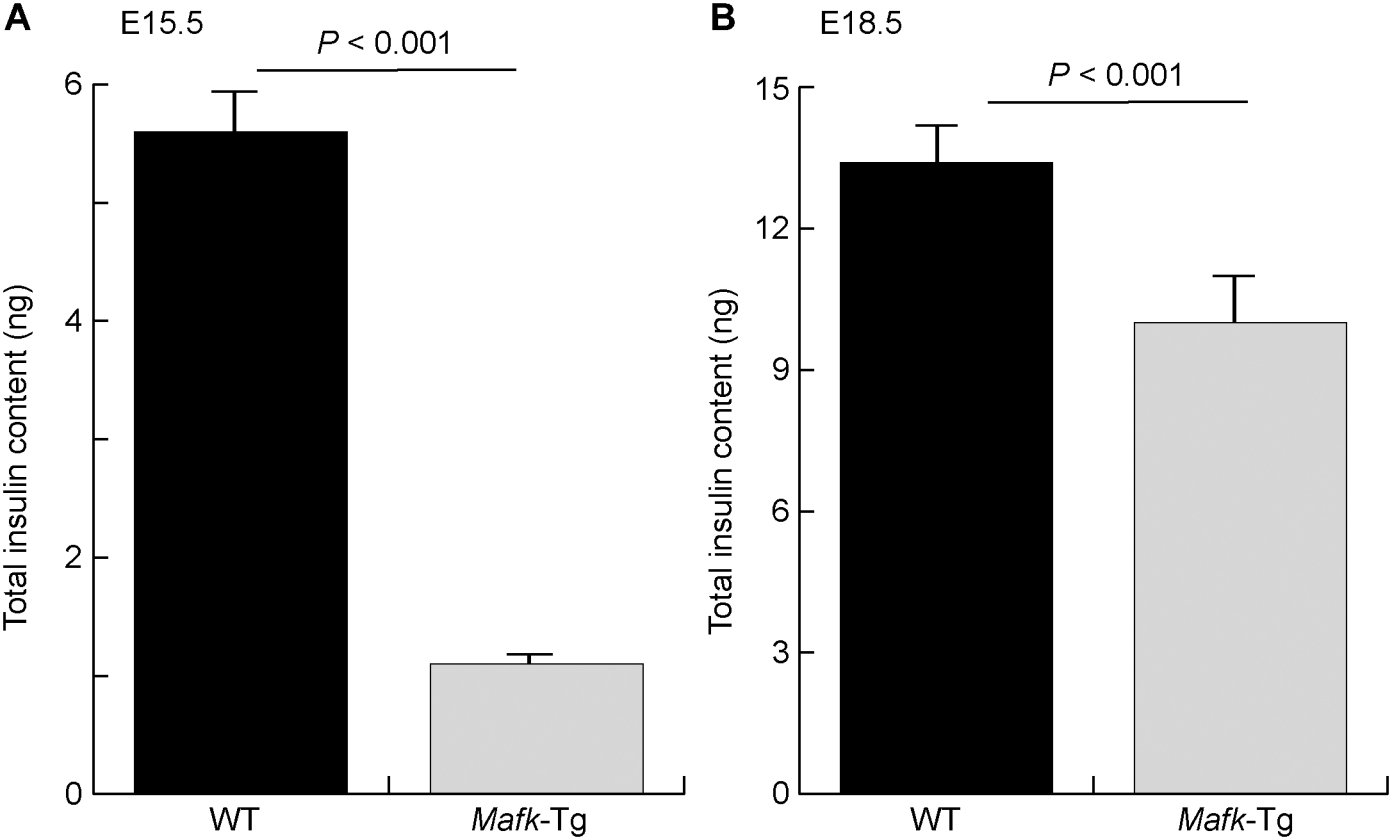
The changes in total pancreatic insulin content. The total insulin content of whole pancreata collected from embryos at E15.5 (A) (n: WT = 6; *Mafk-*Tg = 7) and E18.5 (B) (n: WT = 4; *Mafk-*Tg = 7). The error bars represent the standard errors of the means.

To clarify the extent of these changes, we quantified the changes in insulin+ and glucagon+ cell populations based on their numbers. The total numbers of insulin+ and glucagon+ cells were counted in representative sections throughout the whole pancreas of embryos at E18.5. In *Mafk*-Tg embryos, the total count of insulin+ cells was decreased (3.1 ± 0.19 × 10^3 vs. 6.21 ± 0.72 × 10^3 in WT, *P* = 0.002) (Fig 1B). Conversely, we found the number glucagon+ cells to be significantly increased (3.4 ± 0.14 × 10^3 vs. 2.4 ± 0.20 × 10^3 in WT, *P* = 0.008) (Fig 1C). The total insulin contents were also decreased in *Mafk*-Tg embryos both at E15.5 and at E18.5, consistent with the immunohistochemistry results (Fig 2). The reduction in the total insulin contents was more dramatic at E15.5 (1.1 ± 0.1 ng in Tg vs. 5.6 ± 0.6 ng in WT, *P* < 0.001) compared to E18.5 (10.0 ± 0.39 ng in Tg vs. 13.4 ± 0.33 ng in WT, *P* < 0.001).

### β-cell-specific *Mafk* overexpression altered the gene expression of both β- and α-cell-related factors

**Fig 3:**
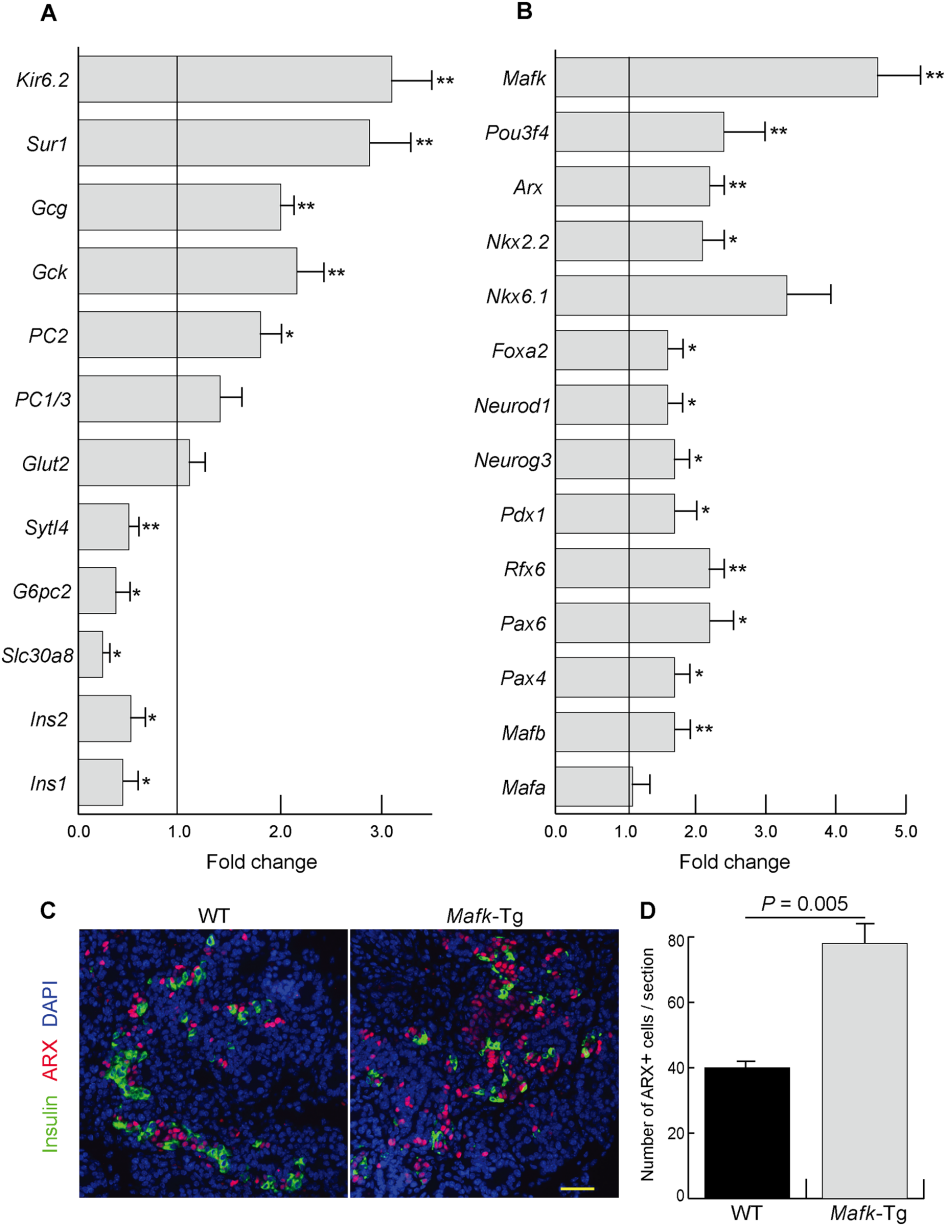
Gene expression in *Mafk-*Tg pancreases. (A, B) The mRNA expression of the indicated genes in the pancreas of *Mafk*-Tg relative to WT at E15.5 (n = 6 per each) *P < 0.05, **P < 0.01. The error bars represent the standard errors of the means. (A) Genes involved in hormone processing and secretion. (B) Transcription factors related to the endocrine development. (C) Immunohistochemical staining of Insulin and ARX of WT and *Mafk*-Tg mice at E15.5. Scale bars = 40 μm. (D) The average number of cells that appeared positive for ARX immunostaining per pancreatic section at E15.5 (n: WT = 3, *Mafk*-Tg = 3).

Quantitative RT-PCR was performed using total RNAs from pancreata of WT and *Mafk*-Tg embryos (n = 6 each) at E15.5. The analysis revealed that the mRNA expression of *Ins1* (0.47 ± 0.07 fold, *P* = 0.03), *Ins2* (0.52 ± 0.07 fold, *P* = 0.04), *Slc30a8* (0.25 ± 0.02 fold, *P* = 0.02), *G6pc2* (0.37 ± 0.08 fold, *P* = 0.03), and *Sytl4* (0.50 ± 0.05 fold, *P* = 0.001) were decreased in *Mafk*-Tg mice (Fig 3A). As we expected, the expression of these downregulated genes is known to be controlled by large Maf genes [14, 17]. The expression of other β-cell-related genes were either unchanged (*Glut2* (1.1 ± 0.1 fold, *P* = 0.08) and *PC1/3* (1.4 ± 0.2 fold, *P* = 0.20)) or increased (*PC2* (1.8 ± 0.2 fold, *P* = 0.02), *Gck* (2.2 ± 0.5 fold, *P* = 0.005), *Sur1* (2.9 ± 0.4 fold, *P* = 0.003), and *Kir6*.*2* (3.1 ± 0.4 fold, *P* = 0.004)) (Fig 3A). We also examined the key transcription factors related to the endocrine development in *Mafk*-Tg embryos. Unexpectedly, many β-cell-related transcription factors as well as α-cell-related factors were found to be significantly increased in *Mafk*-Tg mice, suggesting that the compensatory mechanisms to maintain normal β-cell numbers have been activated (Fig 3B). These factors included *Mafb* (1.7 ± 0.1 fold, *P* = 0.01), *Pax4* (1.7 ± 0.1 fold, *P* = 0.03), *Pax6* (2.4 ± 0.4 fold, *P* = 0.02), *Rfx6* (2.2 ± 0.1 fold, *P* = 0.01), *Pdx1* (1.7 ± 0.2 fold, *P* = 0.03), *Foxa2* (1.6 ± 0.1 fold, *P* = 0.03), *Ngn3* (1.7 ± 0.1 fold, *P* = 0.03), *Neurod1* (1.6 ± 0.1 fold, *P* = 0.05), *Nkx2*.*2* (2.1 ± 0.2 fold, *P* = 0.01), *Nkx6*.*1* (3.3 ± 0.6 fold, *P* = 0.09), *Arx* (2.1 ± 0.2 fold, *P* = 0.001), and *Pou3f4* (2.4 ± 0.2 fold, *P* = 0.005). Furthermore, immunohistochemistry showed that ARX expression, a master regulator of α-cell development, was increased mainly in the noninsulin-positive cells of *Mafk*-Tg mice (Fig 3C). The latter finding was confirmed by quantitative assessment of the number of ARX+ cells per pancreatic section in both WT (40 ± 1.2) and *Mafk*-Tg mice (78 ± 6.6, *P* = 0.005) (Fig 3D).

### MAFK inhibited the activation of insulin promoter

**Fig 4:**
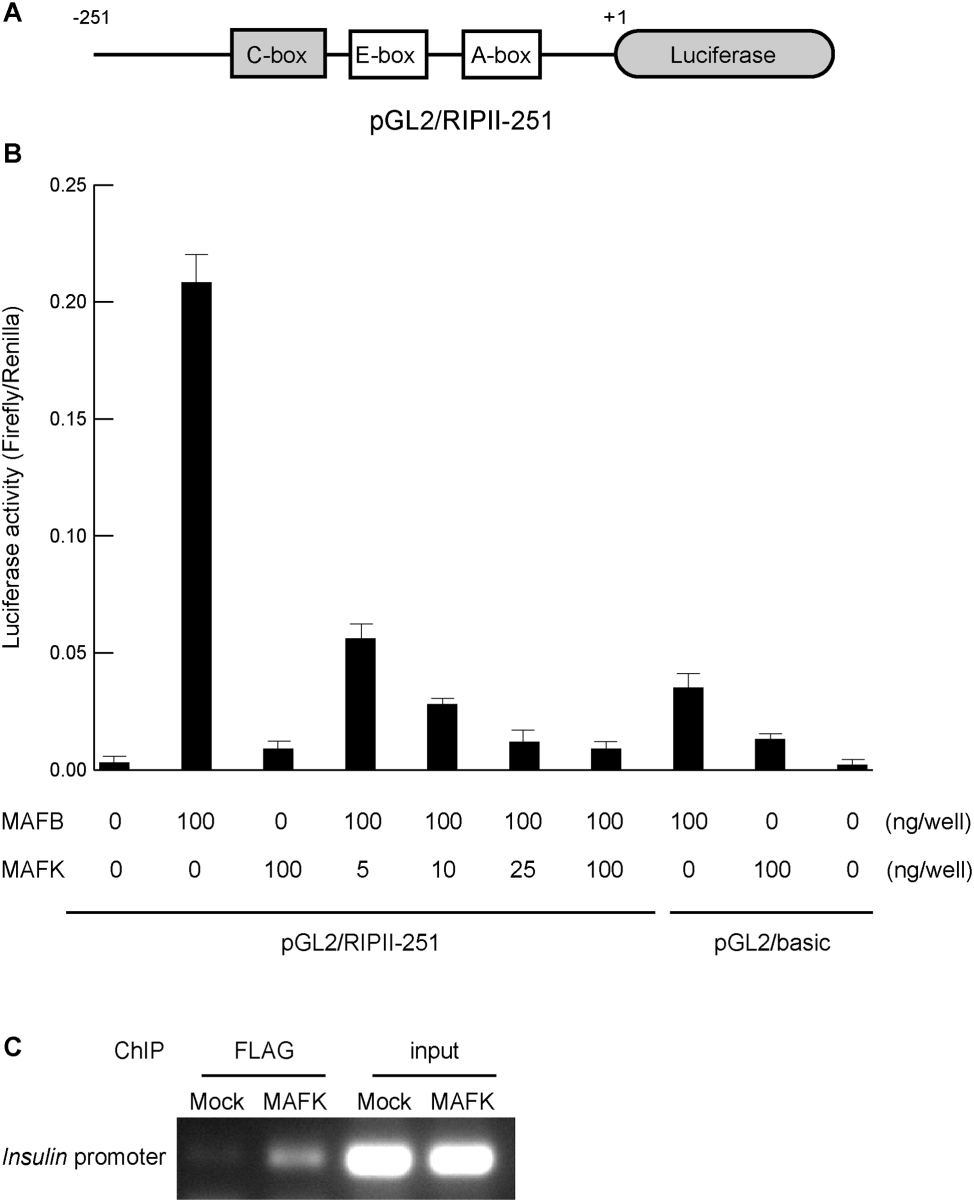
MAFK inhibited the activation of insulin promoter. (A) Schema of the rat insulin promoter II reporter plasmid (pGL2/RIPII-251). (B) *Mafb* and *Mafk* expression plasmids and the pGL2/RIPII-251 plasmid were transfected into NIH3T3 cells. The amount of *Mafk*-expression plasmid were serially increased from 0 to 100 ng. Three independent experiments were conducted and the error bars represent the standard errors of the means. (C) Chromatin immunoprecipitation (ChIP) using anti-FLAG antibody detected binding of MAFK to the *Insulin* promoter region including C-box in NMuMG cells transfected with pCAGIP-FLAG-MAFK. A representative figure of two independent experiments is shown.

The ability of MAFK to suppress the activation of the insulin promoter was examined using luciferase assay. A reporter plasmid containing the luciferase gene under the control of the rat insulin 2 promoter (pGL2/RIPII-251) was used, as previously described [24](Fig 4A). NIH3T3 cells were transfected with MAFK and MAFB expression plasmids with a reporter plasmid, and the luciferase activity was monitored 48 hours after transfection (Fig 4B). MAFB activated the insulin promoter more than 100-fold. In the presence of increased amounts of MAFK, the enhanced activity of insulin promoter by MAFB showed a dose-dependent reduction. These findings indicate that MAFK inhibited MAFB binding to the C-box of the insulin promoter. Chromatin immunoprecipitation (ChIP) using FLAG antibody against NMuMG cells treated with either pCAGIP-FLAG-MAFK or mock plasmids further confirmed these observations (Fig 4C). The binding of MAFK directly to the insulin promoter was evident upon analysis of the immunoprecipitated protein-DNA complex with specific PCR primers.

### No evidence of α-cell transdifferentiation in *Mafk*-Tg β-cells

**Fig 5:**
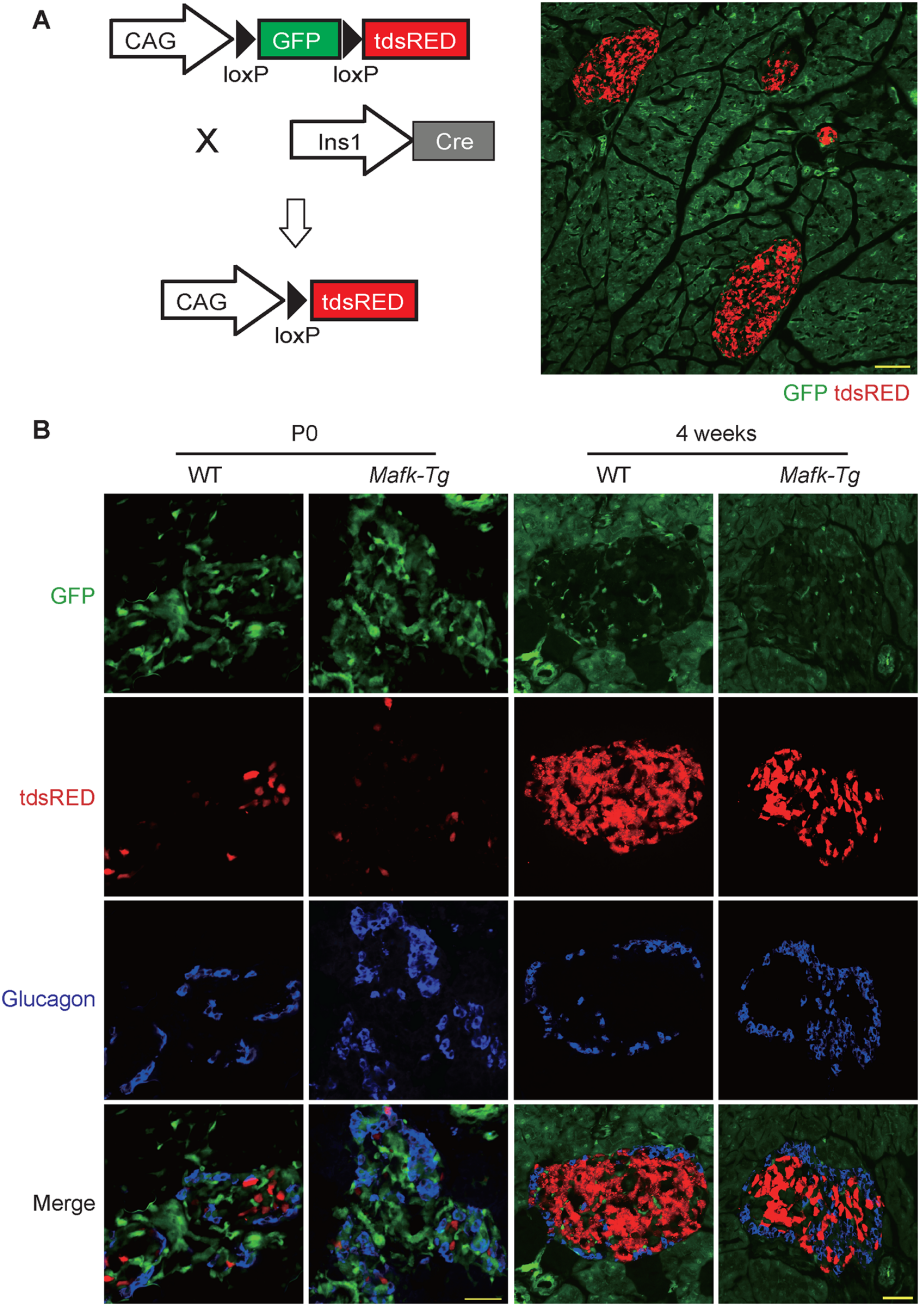
Lineage tracing analysis of β-cell fate in *Mafk-*Tg mice. (A) Strategy of the mice crossing. For β-cell labeling, *Ins1*-Cre;R26GRR male mice were mated with *Mafk*-Tg females. The ability of the reporter mice to express tdsRED exclusively in pancreatic islets was confirmed by microscopic examination of unstained pancreas sections at 4 weeks of age. Scale bar = 100 μm. (B) Higher number of glucagon+ cells was detected in *Mafk*-Tg, however no cells coexpressing tdsRED and glucagon were observed at neither P0 nor 4W (n = 3 per each genotype). Scale bars = 40 μm.

The first observation of the reversion of the absolute number of insulin+ and glucagon+ cells in *Mafk*-Tg mice raised the possibility that β-cell-specific *Mafk* overexpression could induce their transdifferentiation to α-cells during endocrine development. To answer this question, we labeled the β-cell lineage using *Ins1*-Cre;R26GRR mice, which express GFP ubiquitously before and tdsRed exclusively in β cells after Cre recombination (Fig 5A). We collected 8 sections at 100-μm intervals apart from *Mafk*-Tg;*Ins1*-Cre;R26GRR and control WT;*Ins1*-Cre25;R26GRR (n = 3 per mouse) at P0 and 4 weeks of age and stained them with glucagon antibody in order to examine the adult α-cells that might be derived from the β-cell lineage. Although higher numbers of glucagon+ cells were detected in *Mafk*-Tg mice at 4 weeks of age, glucagon+ tdsRed+ cells were observed in 0.52% of the total glucagon+ cells in *Mafk*-Tg mice (n = 763) and these cells were 0.66% of the total glucagon+ cells in the controls (n = 752) (Fig 5B). These observations suggest that the increased number of α-cells in *Mafk*-Tg mice might be due to other causes rather than β-cell transdifferentiation.

### *Mafk* overexpression suppressed β-cell proliferation

**Fig 6:**
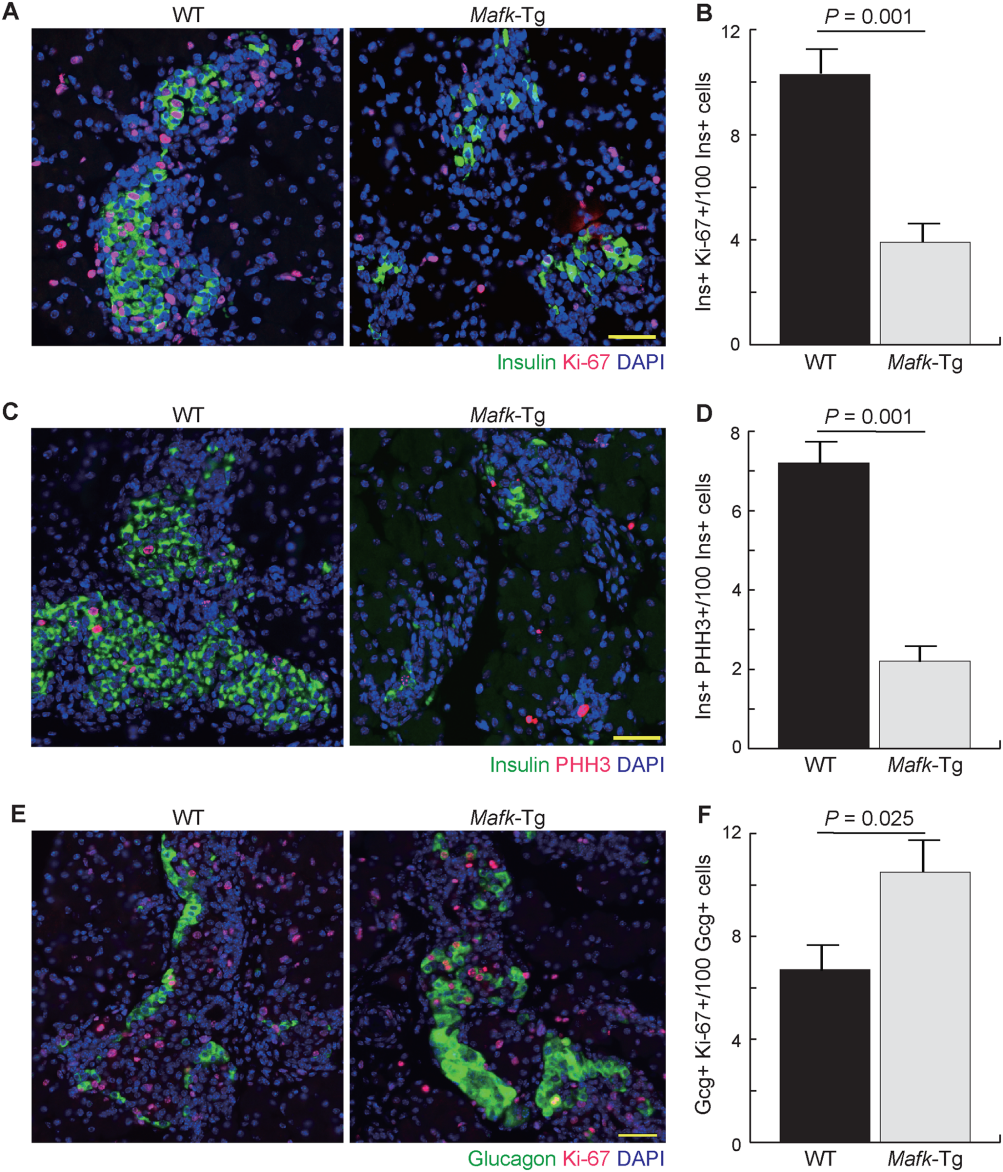
Cell proliferation assay in *Mafk*-Tg mice. (A) Immunohistochemical analysis of pancreata from WT and *Mafk*-Tg mice using Ki-67 and Insulin antibodies at E18.5. Scale bars = 40 μm. (B) The percentage of cells that appeared double positive for both Ki-67 and insulin (n: WT = 4 (463/4135), *Mafk*-Tg = 5 (112/2893)). (C) Immunohistochemical analysis of pancreata from WT and *Mafk*-Tg mice using pHH3 and Insulin antibodies at E18.5. Scale bars = 40 μm. (D) The percentage of the cells that appeared as double positive for both pHH3 and Insulin (n: WT = 4 (158/2291), *Mafk*-Tg = 4 (21/957)). (E) Immunohistochemical analysis of pancreata from WT and *Mafk*-Tg mice using Ki-67 and glucagon antibodies at E18.5. Scale bars = 40 μm. (F) The percentage of proliferating α-cell that appeared as double positive for both Ki-67 and Glucagon (n: WT = 3 (31/429), *Mafk*-Tg = 4 (76/746)). The error bars represent the standard errors of the means.

To examine the reason for reduction in β-cell numbers during the prenatal and early postnatal periods, we performed immunohistochemical analysis using the cell cycle marker Ki-67 (Fig 6A). Six different sections from WT and *Mafk*-Tg embryos (n = 3 per embryo) were collected at 100-μm intervals and stained using Ki-67 and insulin antibodies. The number of double positive cells was counted and divided by the total number of insulin+ cells to estimate the percentage of proliferating β-cells. The result showed that the percentage of proliferating β-cells was lower in *Mafk*-Tg mice (3.9 ± 0.31%) than in WT mice (10.3 ± 0.91%, *P* = 0.001) at E18.5 (Fig 6B). This finding was further examined using another cell cycle marker, pHH3 (Fig 6C). Quantitative comparison of the percentage of the cells that appeared as double positive for both pHH3 and insulin (2.2 ± 0.4% in Tg vs. 7.2 ± 0.6% in WT, *P* = 0.001) was in line with that obtained from Ki-67 staining, suggesting that β-cell proliferation is reduced or delayed in *Mafk*-Tg mice (Fig 6D). Impaired proliferation of blood cells induced by *Mafk* overexpression is also reported *in vivo*, suggesting that excess amounts of MAFK has an antiproliferative effect regardless of cell type [23, 34].

We also examined the changes in α-cell proliferation using Ki-67 staining and found higher percentage of cells with double immunoreactivity for both glucagon and Ki-67 at E18.5 in *Mafk*-Tg (10.5 ± 0.9%) compared to WT embryos (6.7 ± 0.7%, *P* = 0.025)(n = 3 per embryo) (Fig 6E and 6F). This observation indicated that the increase in the α-cell numbers was mainly due to their proliferation rather than β-cell dedifferentiation.

### Microarray analysis of pancreas from *Mafk*-Tg embryos revealed differential expression of islet-related genes

**Fig 7:**
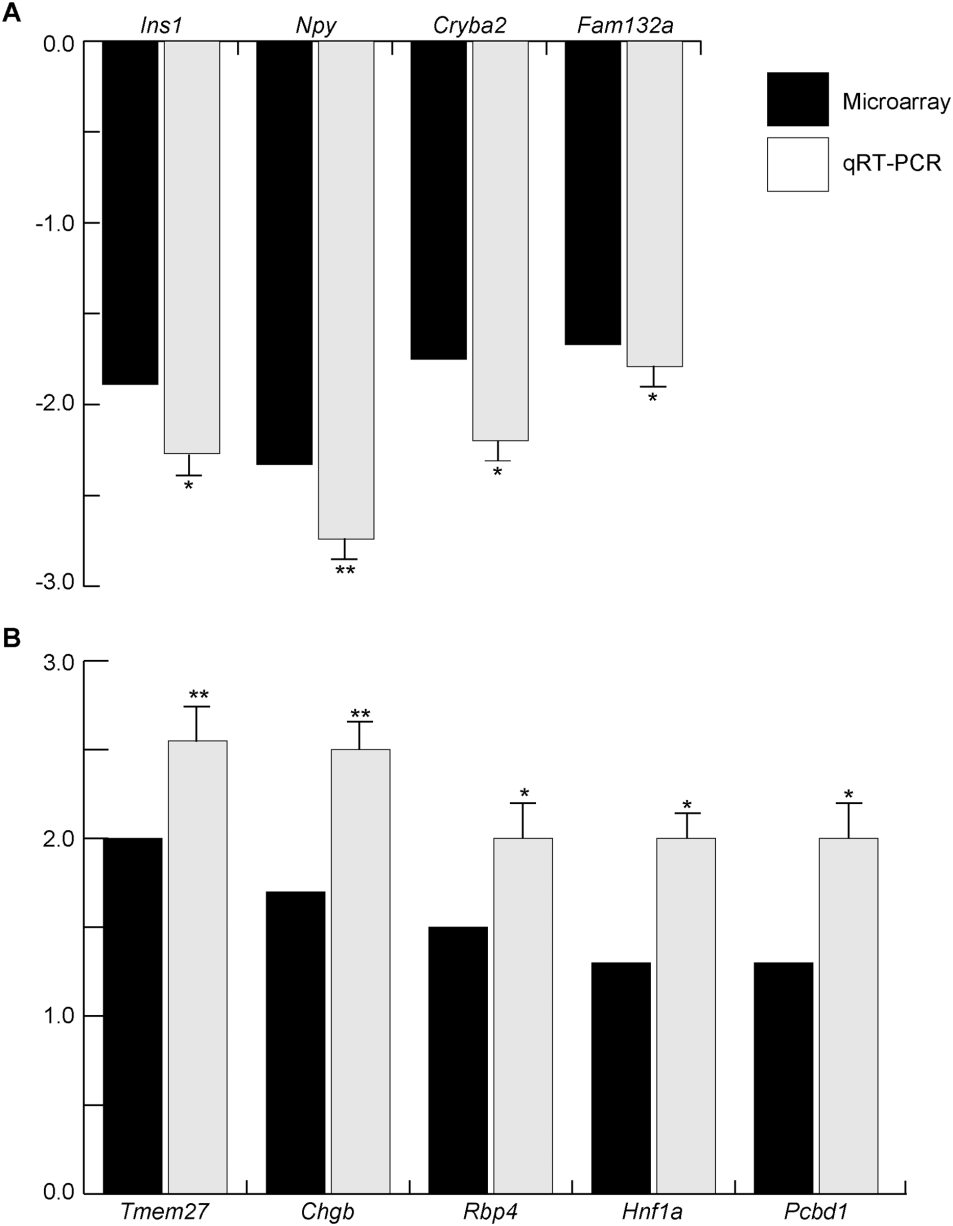
Validation of microarray data by qRT-PCR in WT and *Mafk*-Tg pancreata at E15.5. The expression levels of microarray and qRT-PCR are shown in black and gray columns, respectively. The *Mafk*-Tg expression (n = 8) of indicated genes was shown as relative to their expression in WT (n = 4). *P < 0.05, **P < 0.01. The error bars represent the standard errors of the means. (A) Downregulated genes. (B) Upregulated genes.

To identify genes potentially involved in endocrine development and function, we conducted unsupervised microarray analysis using total RNA from pancreata of WT and *Mafk*-Tg embryos collected at E15.5. qRT-PCR analyses, as shown in Fig 3, indicated that novel genes involved in β-cell functions are expected to show differential expression between genotypes. After data normalization and probe summarization, a list of 554 upregulated and 548 downregulated probe sets was generated (S1 Table). Due to the limited sample size (n = 3 per genotype) and the fact that there is a low abundance of endocrine cells within the pancreas during this stage of development, the cutoff was set at 1.2-fold change [35, 36]. Sixteen genes (namely *Ins1*, *Ins2*, *Slc30a8*, *G6pc2*, *Sytl4*, *Gcg*, *Mafb*, *Pax4*, *Pax6*, *Rfx6*, *Pdx1*, *Foxa2*, *Ngn3*, *Neurod1*, *Nkx2*.*2* and *Pou3f4*) from a total of 22 genes (72.7%) that show significant difference (Fig 3) were found to be included in the up- and down-regulated gene list, suggesting that microarray can be used for further analysis. In order to identify any uncharacterized genes that might be involved in endocrine development and function, we chose several candidate genes for qRT-PCR analyses. For that experiment we used individual biological samples (n = 4–8 per experiment) to confirm the changes observed with the technical replicates that were used for microarray analysis. For qRT-PCR, we chose several transcripts that we thought to be potential factors involved in endocrine development and function, some of which had not been fully characterized (Fig 7). In addition to *Ins1*, we identified 3 novel candidate genes that showed a significant reduction of gene expression in *Mafk*-Tg, namely *Npy*, *Cryba2*, and *Fam132a* (Fig 7A). Additionally, from the upregulated gene group, the expression of *Tmem27*, *Chgb*, *Rbp4*, *Hnf1a* and *Pcbd1* was confirmed (Fig 7B). These results suggest that the gene expression profile of *Mafk*-Tg pancreata can provide a unique set of novel genes that possibly play various roles in endocrine development and function.

## Discussion

In this study, we were able to detect insulin/glucagon double positive cells, which are considered to be endocrine progenitors, in *Mafa*^−/−^, *Mafk*-Tg, and *Mafa*^−/−^;*Mafk*-Tg embryos as well as WT embryos at E12.5. Our findings indicate that neither *Mafa* deficiency nor β-cell-specific *Mafk* overexpression have any deleterious effect on the development of the early pancreatic progenitors. The islets of the *Mafa*^−/−^ mice also showed no marked structural changes when compared to WT mice during the whole prenatal life, consistent with a previous study using *Mafa*^Δpanc^ mice [17]. In contrast, as observed in *Mafk*-Tg and *Mafa*^−/−^;*Mafk*-Tg mice, overexpression of *Mafk* impaired the islet morphogenesis at the late gestational stage, with up to 50% reduction in the number of β-cells and total insulin content. This finding corresponds with that of systemic *Mafb*-deficient embryos in regard to β-cell development [15]. The phenotype observed in *Mafk*-Tg mice probably represents a part of conditional deletion of *Mafb* in β-cells. On this point, we could not rule out the direct role of overexpressed MAFK on other differentiation factors in a large MAF independent manner. However, the increasing expression of *Mafa* in neonatal β-cells probably restored the islet structure in *Mafk*-Tg mice by 5 weeks of age, suggesting that *Mafk* overexpression primarily blocked the large MAF function in embryos [37]. This idea is also supported by a recent report showing the opposing effect of MAFA and small MAFs on the insulin promoter [38].

*Mafk*-deficient mice do not exhibited any abnormalities [19]. β-cell specific transgenic mice, which express a dominant negative form of MAFK (DN-MAFK), show normal glucose tolerance under normal chow diet [38]. In contrast, under high fat diet conditions, DN-MAFK overexpression improves insulin secretion and glucose metabolism. Together with the observation that high fat diet feeding increases small MAF expression in β-cells, MAFK probably downregulates the expression of genes regulated by MAFA under stressed conditions partly due to the blocking of MAFA binding to MARE on the target [38].

Several lines of evidence suggest the capacity of terminally differentiated cells in the endocrine pancreas to lose their integrity and thereby adopt another cell identity [39]. Forced expression of *Arx* in mature β-cells can induce their conversion into a glucagon-positive α-cell phenotype [40]. Moreover β-cells convert into α-cells following deletion of *Dnmt1* or *FoxO1* [41] [42]. Based on these observations, we hypothesized that *Mafk* overexpression might trigger β-cell transdifferentiation/conversation into α-cells. However, as shown in Fig 5, we were not able to find any cells coexpressing tdsRED and glucagon using β-cell lineage tracing, indicating that the increase in α-cells of *Mafk*-Tg mice is likely not to be due to β- to α-cell transdifferentiation, but rather due to the increased proliferation of α-cells. Recently, it is proposed that derepression of *Mafb* in β-cells activates β- to α-cell reprogramming in the absence of *Pdx1* or *Mafa* [43] [44]. Because overexpressed MAFK blocks MAFB function and probably does not initiate the reprogramming cascade in *Mafk*-Tg β-cells, the increase in α-cell numbers as well as α-cell transcription factors might be due to a loss of insulin action [45]. Alternatively, disruption of key β-cell transcription factors (*Mafs* or *Pdx1*) during the early postnatal period does not cause β- to α- transdifferentiation (this manuscript and [46]), which occurs after 4 weeks of age [43] [44].

Finally, our analyses of microarray and subsequent qRT-PCR revealed that the downregulated gene group included a set of important transcripts for β-cell function. *Npy*, *Fam132a*, and *Cryba2*, as well as *Ins1* were encountered as potential β-cell-related genes. NPY (Neuropeptide Y) has pleiotropic functions in various tissues including hypothalamus, autonomic nervous system, and adipose tissue. In pancreas, its expression is observed in β-cells during the secondary transition to neonatal period [47, 48]. A previous report showing that NPY treatment of mouse islets significantly enhanced β-cell replication supports our finding of a reduction of β-cell proliferation in *Mafk*-Tg mice [49]. *Fam132a* (also called *Adipolin*) is a novel adipokine associated with roles in glycemic control and insulin sensitization [50]. In addition to adipose tissue, *Fam132a* is thought to be secreted from mouse islets, according to the T1D base (https://www.t1dbase.org/page/Welcome/display). Notably, *Cryba2* is identified as an enriched gene in developing and adult pancreas and its expression is affected by *Ngn3*-deficiency during development, although no clear phenotype in pancreas of ENU-induced *Cryba2* mutants has been reported [51–53].

From the upregulated gene group, TMEM27 plays a role in controlling insulin exocytosis by regulating the soluble N-ethylmaleimide-sensitive factor attachment protein receptor (SNARE) complex assembly [54, 55]. Retinol binding protein 4 (RBP4), which is a principle carrier of blood retinol, contributes insulin resistance in mice and humans [56]. Interestingly, both *Tmem27* and *Rbp4* is upregulated in pancreas from *Mafb*^*-/-*^ during late embryonic period, implying that *Mafk-*Tg mice and *Mafb*^*-/-*^ mice share common gene expression patterns as well as phenotypic similarities [17]. Chromogranin-B (CHGB) is a secretory glycoprotein co-stored with insulin and is found to control the rapid initial phase of insulin secretion [57]. Hepatocyte nuclear factor 1 alpha (HNF1A) controls many genes related to β-cell differentiation, and gene mutations are the most common cause of maturity-onset diabetes of the young [58]. Pterin-4-alpha-carbinolamine dehydratase (PCBD1) is a novel protein that acts as a cofactor for HNF1A-dependent transcription protein, and it is reported that *PCBD1* mutations cause early-onset nonautoimmune diabetes with features similar to dominantly inherited HNF1A-diabetes [59].

In conclusion, β-cell-specific *Mafk* overexpression resulted in impairment in endocrine development through alteration of the expression of many important genes for endocrine development and function. Since *Mafk* overexpression can mimic the targeted disruption of many genes containing MARE sites in their regulatory regions to which MAFK homodimers can bind, our microarray analysis of *Mafk*-Tg embryos provides a unique data set for investigating novel factors that might have possible roles in β-cell development, function, and survival.

## Supporting Information

S1 Table List of the up- and down-regulated genes in the pancreas of *Mafk*-Tg embryos at E15.5. (XLSX)

## References

[1] American Diabetes A. Diagnosis and classification of diabetes mellitus. Diabetes care. 2014;37 Suppl 1:S81–90. 10.2337/dc14-S081 . 24357215

[2] Vetere A, Choudhary A, Burns SM, Wagner BK. Targeting the pancreatic beta-cell to treat diabetes. Nature reviews Drug discovery. 2014;13(4):278–89. 10.1038/nrd4231 . 24525781

[3] Jensen J, Heller RS, Funder-Nielsen T, Pedersen EE, Lindsell C, Weinmaster G, et al Independent development of pancreatic alpha- and beta-cells from neurogenin3-expressing precursors: a role for the notch pathway in repression of premature differentiation. Diabetes. 2000;49(2):163–76. . 10868931 10.2337/diabetes.49.2.163

[4] Gradwohl G, Dierich A, LeMeur M, Guillemot F. neurogenin3 is required for the development of the four endocrine cell lineages of the pancreas. Proceedings of the National Academy of Sciences of the United States of America. 2000;97(4):1607–11. 10677506 10.1073/pnas.97.4.1607 PMC26482

[5] Gu G, Dubauskaite J, Melton DA. Direct evidence for the pancreatic lineage: NGN3+ cells are islet progenitors and are distinct from duct progenitors. Development. 2002;129(10):2447–57. . 11973276 10.1242/dev.129.10.2447

[6] Desgraz R, Herrera PL. Pancreatic neurogenin 3-expressing cells are unipotent islet precursors. Development. 2009;136(21):3567–74. 10.1242/dev.039214 19793886 PMC2761107

[7] Slack JM. Developmental biology of the pancreas. Development. 1995;121(6):1569–80. . 7600975 10.1242/dev.121.6.1569

[8] Benitez CM, Goodyer WR, Kim SK. Deconstructing pancreas developmental biology. Cold Spring Harbor perspectives in biology. 2012;4(6). 10.1101/cshperspect.a012401 22587935 PMC3367550

[9] Nishizawa M, Kataoka K, Goto N, Fujiwara KT, Kawai S. v-maf, a viral oncogene that encodes a "leucine zipper" motif. Proceedings of the National Academy of Sciences of the United States of America. 1989;86(20):7711–5. 2554284 10.1073/pnas.86.20.7711 PMC298140

[10] Kataoka K, Noda M, Nishizawa M. Maf nuclear oncoprotein recognizes sequences related to an AP-1 site and forms heterodimers with both Fos and Jun. Molecular and cellular biology. 1994;14(1):700–12. 8264639 10.1128/mcb.14.1.700-712.1994 PMC358419

[11] Eychene A, Rocques N, Pouponnot C. A new MAFia in cancer. Nature reviews Cancer. 2008;8(9):683–93. 10.1038/nrc2460 . 19143053

[12] Hang Y, Stein R. MafA and MafB activity in pancreatic beta cells. Trends in endocrinology and metabolism: TEM. 2011;22(9):364–73. 10.1016/j.tem.2011.05.003 21719305 PMC3189696

[13] Matsuoka TA, Artner I, Henderson E, Means A, Sander M, Stein R. The MafA transcription factor appears to be responsible for tissue-specific expression of insulin. Proceedings of the National Academy of Sciences of the United States of America. 2004;101(9):2930–3. 10.1073/pnas.0306233101 14973194 PMC365722

[14] Nishimura W, Kondo T, Salameh T, El Khattabi I, Dodge R, Bonner-Weir S, et al A switch from MafB to MafA expression accompanies differentiation to pancreatic beta-cells. Developmental biology. 2006;293(2):526–39. 10.1016/j.ydbio.2006.02.028 16580660 PMC2390934

[15] Artner I, Blanchi B, Raum JC, Guo M, Kaneko T, Cordes S, et al MafB is required for islet beta cell maturation. Proceedings of the National Academy of Sciences of the United States of America. 2007;104(10):3853–8. 10.1073/pnas.0700013104 17360442 PMC1803762

[16] Zhang C, Moriguchi T, Kajihara M, Esaki R, Harada A, Shimohata H, et al MafA is a key regulator of glucose-stimulated insulin secretion. Molecular and cellular biology. 2005;25(12):4969–76. 10.1128/MCB.25.12.4969-4976.2005 15923615 PMC1140590

[17] Artner I, Hang Y, Mazur M, Yamamoto T, Guo M, Lindner J, et al MafA and MafB regulate genes critical to beta-cells in a unique temporal manner. Diabetes. 2010;59(10):2530–9. 10.2337/db10-0190 20627934 PMC3279542

[18] Lecoin L, Sii-Felice K, Pouponnot C, Eychene A, Felder-Schmittbuhl MP. Comparison of maf gene expression patterns during chick embryo development. Gene expression patterns: GEP. 2004;4(1):35–46. . 14678826 10.1016/s1567-133x(03)00152-2

[19] Kotkow KJ, Orkin SH. Complexity of the erythroid transcription factor NF-E2 as revealed by gene targeting of the mouse p18 NF-E2 locus. Proceedings of the National Academy of Sciences of the United States of America. 1996;93(8):3514–8. 8622968 10.1073/pnas.93.8.3514 PMC39641

[20] Shavit JA, Motohashi H, Onodera K, Akasaka J, Yamamoto M, Engel JD. Impaired megakaryopoiesis and behavioral defects in mafG-null mutant mice. Genes & development. 1998;12(14):2164–74. 9679061 10.1101/gad.12.14.2164 PMC317009

[21] Onodera K, Shavit JA, Motohashi H, Katsuoka F, Akasaka JE, Engel JD, et al Characterization of the murine mafF gene. The Journal of biological chemistry. 1999;274(30):21162–9. . 10409670 10.1074/jbc.274.30.21162

[22] Katsuoka F, Motohashi H, Ishii T, Aburatani H, Engel JD, Yamamoto M. Genetic evidence that small maf proteins are essential for the activation of antioxidant response element-dependent genes. Molecular and cellular biology. 2005;25(18):8044–51. 10.1128/MCB.25.18.8044-8051.2005 16135796 PMC1234339

[23] Motohashi H, O'Connor T, Katsuoka F, Engel JD, Yamamoto M. Integration and diversity of the regulatory network composed of Maf and CNC families of transcription factors. Gene. 2002;294(1–2):1–12. . 12234662 10.1016/s0378-1119(02)00788-6

[24] Shimohata H, Yoh K, Morito N, Shimano H, Kudo T, Takahashi S. MafK overexpression in pancreatic beta-cells caused impairment of glucose-stimulated insulin secretion. Biochemical and biophysical research communications. 2006;346(3):671–80. 10.1016/j.bbrc.2006.05.184 . 16780794

[25] Shimohata H, Yoh K, Fujita A, Morito N, Ojima M, Tanaka H, et al MafA-deficient and beta cell-specific MafK-overexpressing hybrid transgenic mice develop human-like severe diabetic nephropathy. Biochemical and biophysical research communications. 2009;389(2):235–40. 10.1016/j.bbrc.2009.08.124 . 19715672

[26] Hasegawa Y, Daitoku Y, Mizuno S, Tanimoto Y, Mizuno-Iijima S, Matsuo M, et al Generation and characterization of Ins1-cre-driver C57BL/6N for exclusive pancreatic beta cell-specific Cre-loxP recombination. Experimental animals / Japanese Association for Laboratory Animal Science. 2014;63(2):183–91. . 24770644 10.1538/expanim.63.183 PMC4160984

[27] Hasegawa Y, Daitoku Y, Sekiguchi K, Tanimoto Y, Mizuno-Iijima S, Mizuno S, et al Novel ROSA26 Cre-reporter knock-in C57BL/6N mice exhibiting green emission before and red emission after Cre-mediated recombination. Experimental animals / Japanese Association for Laboratory Animal Science. 2013;62(4):295–304. 24172193 10.1538/expanim.62.295 PMC4160954

[28] Kitamura K, Yanazawa M, Sugiyama N, Miura H, Iizuka-Kogo A, Kusaka M, et al Mutation of ARX causes abnormal development of forebrain and testes in mice and X-linked lissencephaly with abnormal genitalia in humans. Nature genetics. 2002;32(3):359–69. 10.1038/ng1009 . 12379852

[29] Kajihara M, Sone H, Amemiya M, Katoh Y, Isogai M, Shimano H, et al Mouse MafA, homologue of zebrafish somite Maf 1, contributes to the specific transcriptional activity through the insulin promoter. Biochemical and biophysical research communications. 2003;312(3):831–42. 10.1016/j.bbrc.2003.10.196 . 14680841

[30] Okita Y, Kamoshida A, Suzuki H, Itoh K, Motohashi H, Igarashi K, et al Transforming growth factor-beta induces transcription factors MafK and Bach1 to suppress expression of the heme oxygenase-1 gene. The Journal of biological chemistry. 2013;288(28):20658–67. 10.1074/jbc.M113.450478 23737527 PMC3711329

[31] Hashimoto T, Kawano H, Daikoku S, Shima K, Taniguchi H, Baba S. Transient coappearance of glucagon and insulin in the progenitor cells of the rat pancreatic islets. Anatomy and embryology. 1988;178(6):489–97. . 2464956 10.1007/BF00305036

[32] De Krijger RR, Aanstoot HJ, Kranenburg G, Reinhard M, Visser WJ, Bruining GJ. The midgestational human fetal pancreas contains cells coexpressing islet hormones. Developmental biology. 1992;153(2):368–75. . 1356859 10.1016/0012-1606(92)90121-v

[33] Eto K, Nishimura W, Oishi H, Udagawa H, Kawaguchi M, Hiramoto M, et al MafA is required for postnatal proliferation of pancreatic beta-cells. PloS one. 2014;9(8):e104184 10.1371/journal.pone.0104184 25126749 PMC4134197

[34] Yoh K, Sugawara T, Motohashi H, Takahama Y, Koyama A, Yamamoto M, et al Transgenic over-expression of MafK suppresses T cell proliferation and function in vivo. Genes to cells: devoted to molecular & cellular mechanisms. 2001;6(12):1055–66. . 11737266 10.1046/j.1365-2443.2001.00489.x

[35] Wilding Crawford L, Tweedie Ables E, Oh YA, Boone B, Levy S, Gannon M. Gene expression profiling of a mouse model of pancreatic islet dysmorphogenesis. PloS one. 2008;3(2):e1611 10.1371/journal.pone.0001611 18297134 PMC2249940

[36] Rubel CA, Lanz RB, Kommagani R, Franco HL, Lydon JP, DeMayo FJ. Research resource: Genome-wide profiling of progesterone receptor binding in the mouse uterus. Molecular endocrinology. 2012;26(8):1428–42. 10.1210/me.2011-1355 22638070 PMC3404303

[37] Aguayo-Mazzucato C, Koh A, El Khattabi I, Li WC, Toschi E, Jermendy A, et al Mafa expression enhances glucose-responsive insulin secretion in neonatal rat beta cells. Diabetologia. 2011;54(3):583–93. 10.1007/s00125-010-2026-z 21190012 PMC3047400

[38] Nomoto H, Kondo T, Miyoshi H, Nakamura A, Hida Y, Yamashita K, et al Inhibition of Small Maf Function in Pancreatic beta-Cells Improves Glucose Tolerance Through the Enhancement of Insulin Gene Transcription and Insulin Secretion. Endocrinology. 2015;156(10):3570–80. 10.1210/en.2014-1906 25763640 PMC4588816

[39] Thorel F, Nepote V, Avril I, Kohno K, Desgraz R, Chera S, et al Conversion of adult pancreatic alpha-cells to beta-cells after extreme beta-cell loss. Nature. 2010;464(7292):1149–54. 10.1038/nature08894 20364121 PMC2877635

[40] Collombat P, Hecksher-Sorensen J, Krull J, Berger J, Riedel D, Herrera PL, et al Embryonic endocrine pancreas and mature beta cells acquire alpha and PP cell phenotypes upon Arx misexpression. The Journal of clinical investigation. 2007;117(4):961–70. 10.1172/JCI29115 17404619 PMC1839241

[41] Dhawan S, Georgia S, Tschen SI, Fan G, Bhushan A. Pancreatic beta cell identity is maintained by DNA methylation-mediated repression of Arx. Developmental cell. 2011;20(4):419–29. 10.1016/j.devcel.2011.03.012 21497756 PMC3086024

[42] Talchai C, Xuan S, Lin HV, Sussel L, Accili D. Pancreatic beta cell dedifferentiation as a mechanism of diabetic beta cell failure. Cell. 2012;150(6):1223–34. 10.1016/j.cell.2012.07.029 22980982 PMC3445031

[43] Gao T, McKenna B, Li C, Reichert M, Nguyen J, Singh T, et al Pdx1 maintains beta cell identity and function by repressing an alpha cell program. Cell metabolism. 2014;19(2):259–71. 10.1016/j.cmet.2013.12.002 24506867 PMC3950964

[44] Nishimura W, Takahashi S, Yasuda K. MafA is critical for maintenance of the mature beta cell phenotype in mice. Diabetologia. 2015;58(3):566–74. 10.1007/s00125-014-3464-9 . 25500951

[45] Liu Z, Kim W, Chen Z, Shin YK, Carlson OD, Fiori JL, et al Insulin and glucagon regulate pancreatic alpha-cell proliferation. PloS one. 2011;6(1):e16096 10.1371/journal.pone.0016096 21283589 PMC3026810

[46] Gannon M, Ables ET, Crawford L, Lowe D, Offield MF, Magnuson MA, et al pdx-1 function is specifically required in embryonic beta cells to generate appropriate numbers of endocrine cell types and maintain glucose homeostasis. Developmental biology. 2008;314(2):406–17. 10.1016/j.ydbio.2007.10.038 18155690 PMC2269701

[47] Myrsen-Axcrona U, Ekblad E, Sundler F. Developmental expression of NPY, PYY and PP in the rat pancreas and their coexistence with islet hormones. Regulatory peptides. 1997;68(3):165–75. . 9100283 10.1016/s0167-0115(96)02113-1

[48] Whim MD. Pancreatic beta cells synthesize neuropeptide Y and can rapidly release peptide co-transmitters. PloS one. 2011;6(4):e19478 10.1371/journal.pone.0019478 21559341 PMC3084883

[49] Cho YR, Kim CW. Neuropeptide Y promotes beta-cell replication via extracellular signal-regulated kinase activation. Biochemical and biophysical research communications. 2004;314(3):773–80. . 14741702 10.1016/j.bbrc.2003.12.170

[50] Enomoto T, Ohashi K, Shibata R, Higuchi A, Maruyama S, Izumiya Y, et al Adipolin/C1qdc2/CTRP12 protein functions as an adipokine that improves glucose metabolism. The Journal of biological chemistry. 2011;286(40):34552–8. 10.1074/jbc.M111.277319 21849507 PMC3186379

[51] Petri A, Ahnfelt-Ronne J, Frederiksen KS, Edwards DG, Madsen D, Serup P, et al The effect of neurogenin3 deficiency on pancreatic gene expression in embryonic mice. Journal of molecular endocrinology. 2006;37(2):301–16. 10.1677/jme.1.02096 . 17032746

[52] Hoffman BG, Zavaglia B, Witzsche J, Ruiz de Algara T, Beach M, Hoodless PA, et al Identification of transcripts with enriched expression in the developing and adult pancreas. Genome biology. 2008;9(6):R99 10.1186/gb-2008-9-6-r99 18554416 PMC2481431

[53] Puk O, Ahmad N, Wagner S, Hrabe de Angelis M, Graw J. First mutation in the betaA2-crystallin encoding gene is associated with small lenses and age-related cataracts. Investigative ophthalmology & visual science. 2011;52(5):2571–6. 10.1167/iovs.10-6443 . 21212184

[54] Fukui K, Yang Q, Cao Y, Takahashi N, Hatakeyama H, Wang H, et al The HNF-1 target collectrin controls insulin exocytosis by SNARE complex formation. Cell metabolism. 2005;2(6):373–84. 10.1016/j.cmet.2005.11.003 . 16330323

[55] Akpinar P, Kuwajima S, Krutzfeldt J, Stoffel M. Tmem27: a cleaved and shed plasma membrane protein that stimulates pancreatic beta cell proliferation. Cell metabolism. 2005;2(6):385–97. 10.1016/j.cmet.2005.11.001 . 16330324

[56] Yang Q, Graham TE, Mody N, Preitner F, Peroni OD, Zabolotny JM, et al Serum retinol binding protein 4 contributes to insulin resistance in obesity and type 2 diabetes. Nature. 2005;436(7049):356–62. 10.1038/nature03711 . 16034410

[57] Obermuller S, Calegari F, King A, Lindqvist A, Lundquist I, Salehi A, et al Defective secretion of islet hormones in chromogranin-B deficient mice. PloS one. 2010;5(1):e8936 10.1371/journal.pone.0008936 20126668 PMC2812483

[58] Shields BM, Hicks S, Shepherd MH, Colclough K, Hattersley AT, Ellard S. Maturity-onset diabetes of the young (MODY): how many cases are we missing? Diabetologia. 2010;53(12):2504–8. 10.1007/s00125-010-1799-4 . 20499044

[59] Simaite D, Kofent J, Gong M, Ruschendorf F, Jia S, Arn P, et al Recessive mutations in PCBD1 cause a new type of early-onset diabetes. Diabetes. 2014;63(10):3557–64. 10.2337/db13-1784 . 24848070

